# Optimisation of curvilinear external shading of windows in cellular offices

**DOI:** 10.1371/journal.pone.0203575

**Published:** 2018-09-07

**Authors:** Sanja Stevanović, Dragan Stevanović

**Affiliations:** Mathematical Institute, Serbian Academy of Sciences and Arts, Belgrade, Serbia; University of Science and Technology Beijing, CHINA

## Abstract

Shading of windows influences building cooling and heating loads through control of solar heat gains, and lighting load through access to available daylight. Shading shape thus presents an important factor both in building energy analysis and building aesthetics. Curvilinearity of solar paths suggests that the optimal shading shape may be curvilinear as well, and our aim here is to test this expectation. To accommodate curvilinearity of shading shape, outer edges of shading, which consists of overhang, western and eastern fins, are modeled as non-uniform rational basis spline (NURBS) curves, a widely accepted representation standard for curves in design industry. As a case study, a cellular office is considered in the Pacific Northwest National Laboratory (PNNL) office building model, with its overhang lined up by seven control points, and the fins lined up by five control points each, with two ending control points joint for the overhang and the fins. With control points allowed to take on nine different alternative depths, genetic optimisation is employed for 16 representative USA climates with respect to total equivalent source energy for heating, cooling and lighting loads. The main finding is that in a very close proximity to optimal shadings found by genetic optimisation there exist shadings with much simpler control point structure, obtained by identifying depths of successive control points, that have nearly rectangular overhangs. Since the difference between these simpler shadings and the optimal ones is less than 0.24%, this partially rejects the expectation that the optimal shading shape should be curvilinear. Structure of these near-optimal shadings also suggests a new way to partition shadings into independent regions: the lower and the upper parts of the western fin, joints of the overhang with the western and the eastern fin, the interior part of the overhang and the rest of the eastern fin.

## Introduction

A large part of energy in buildings is used to provide comfortable thermal conditions to their occupants. Shading of windows is a well accepted way of passive reduction of cooling loads, popularised by Le Corbusier in the form of brise-soleils in his Unité d’habitation buildings in the 1950s [1]. Le Corbusier’s goal was that brise-soleils fully shade windows at noon in summer months, but not to block the sun during winter months. This approach led to development of a number of methods for shading design that are based on solar path projections and cut-off days and hours, during which complete shading of windows is required. Early examples include methods of Olgyay and Olgyay [2] and Mazria [3], where Olgyay and Olgyay use a projection of the sun onto a horizontal plane, while Mazria uses a projection onto the vertical cylinder. In both cases the overheating period is plotted onto the solar path diagram which helps to define the shading mask that blocks direct sun radiation during this period. Shaviv [4, 5] developed a computer program that from a solar path generates a set of possible shadings which prevent direct radiation for each month. Arumí-Noé’s algorithm [6] first finds a winter solar funnel surface that ensures full insolation, and then clips it subject to the summer shading conditions. Dubois [7] developed a chart, complementary to Mazria’s solar path diagram, by taking into account additional information on the window solar heat gain coefficient and the incidence angle between the window and the sun beam. Marsh [8] proposed a method that obtains shading shape by projecting solar position at required cut-off dates and hours onto a plane of the shading. Cheung and Chung [9] divided the sky map into small 5° × 5° patches in order to be able to predict probable sunlight duration on windows in a densely packed building environment, which is then used in the design of exterior shading.

Another approach present in the literature consists of dividing shading support surface into smaller cells and processing each cell separately. In his cellular method Kaftan [10] divides the shading surface into two-dimensional array of cells and for each cell calculates the amount of direct solar gains it prevents during time periods for which shading is required. In their Shaderade method, which builds upon the Kaftan’s method, Sargent et al. [11] calculate for each cell and each time period the desired fraction of solar beam energy transmitted through the cell at that time period and then find the optimal transmittance value for a cell from an annual calculation. A welcome characteristics of these two methods is that the cells may be sorted according to calculated values which allows one to identify most and least effective areas of the shading support surface and to collect cells in decreasing order of effectiveness in order to obtain shading with required surface area. However, the shapes suggested by these methods tend to be serrated, so that architect’s interference is necessary to produce aesthetical design.

Nowadays, more than 50 years since Le Corbusier’s seminal buildings, heating loads have become significantly lowered due to standard use of high insulation and tight sealing in construction. In office buildings in milder climates they are even similar in magnitude to lighting loads, due to both high internal gains and minimal illuminance level requirements. Hence it becomes necessary for office shading design methods to take into account all of the heating, cooling and lighting loads. Kaftan and Marsh [12] combined Kaftan’s cellular method with Ecotect in order to predict necessity of shading based on both direct solar gains and thermal comfort indicators. Calculations of desired cell transmittances in Shaderade [11] already include EnergyPlus predictions of heating and cooling loads, but for the base case of a building without shading only. These methods, however, cannot account for lighting load, due to imponderable influence of each particular cell of the shading support structure.

On the other hand, recent shading design methods usually employ genetic algorithms to overcome the difficulty of handling lighting load together with heating and cooling loads. Castorina [13] encodes a shading covering the whole façade with a particle-spring system and employs genetic algorithms and EnergyPlus to optimise a single objective function representing weighted combination of illuminance ratio, lighting load, and the ratio of winter and summer solar gains. Ercan and Elias-Ozkan [14] also consider the whole façade shading by encoding the depth and angle of each shading device and use genetic algorithms and Radiance to optimise daylighting levels, but they do not consider interior thermal conditions. Manzan [15] uses DAYSIM to estimate lighting load and ESP-r to calculate heating and cooling loads, and then use genetic algorithm to determine optimal angle and reveal of a full width rectangular overhang.

Here we are interested in optimal shape of external shading for a south oriented window in a cellular office. As solar path is curvilinear, it is natural to expect that the optimal shading shape will also be curvilinear. To accommodate this curvilinearity, it is assumed that the shading, consisting of an overhang, eastern and western fin, tightly placed around the window, has its outer edges modeled with NURBS curves. Due to their rather general definition, NURBS curves can be used to approximate arbitrary curves, as can be evidenced from a number of earlier works [16–21]. Using NURBS curves here enables sampling the huge space of smooth shading designs by a controllable number of NURBS-based designs, as the shape of NURBS curves is determined by the number of control points and their feasible positions. A genetic algorithm is employed to optimize positions of these control points for representative USA climates with respect to a weighted sum of heating, cooling and lighting loads as a single objective. Discussion is then oriented toward understanding optimisation results that in some cases seem counterintuitive, and toward producing simplified designs with loads close to the optimal ones. Results reported here are continuation of studies initiated in [22, 23].

## Methods

### Cellular office model

The prototype large office building models [24], developed by PNNL and derived from the Department of Energy (DOE) Commercial Reference Building Models, are used as a starting point for this study. These models, whose development and definitions are closely described in [25, 26], represent realistic building characteristics and construction practices, aim to cover more than 70% of the commercial building floor area in the United States for new construction and conform to the American Society of Heating, Refrigerating and Air-Conditioning Engineers (ASHRAE) 90.1-2013 standard. The models are prepared for simulations with EnergyPlus, with version 8.4 used here. They are provided for all main USA climate zones according to the climate zone classification system developed by Briggs et al. [27]. Locations of the PNNL building models, together with a few selected parameters, are listed in Table 1. In addition to USA locations, a model is provided for Vancouver, Canada, as well, which represents the cool, marine climate zone 5C.

**Table 1:** Model locations and selected parameters.

Location	Climate zone	Latitude (°N)	Incident solar radiation rate (W/m^2^)	Window U-value (W/m^2^K)	Window SHGC
Miami	1A: very hot, humid	25.82	121.74	0.60	0.25
Houston	2A: hot, humid	30.00	118.41	0.60	0.25
Phoenix	2B: hot, dry	33.45	167.94	0.60	0.25
Memphis	3A: warm, humid	35.07	129.20	0.55	0.25
El Paso	3B: warm, dry	31.77	162.14	0.55	0.25
San Francisco	3C: warm, marine	37.62	141.94	0.55	0.25
Baltimore	4A: mixed, humid	39.17	129.45	0.42	0.40
Albuquerque	4B: mixed, dry	35.04	168.25	0.42	0.40
Salem	4C: mixed, marine	44.90	116.35	0.42	0.40
Chicago	5A: cool, humid	41.98	122.67	0.42	0.40
Boise	5B: cool, dry	43.62	144.32	0.42	0.40
Vancouver	5C: cool, marine	49.18	111.99	0.42	0.40
Burlington	6A: cold, humid	44.47	118.52	0.42	0.40
Helena	6B: cold, dry	46.60	143.85	0.42	0.40
Duluth	7: very cold	46.83	129.84	0.40	0.45
Fairbanks	8: subarctic	64.82	114.49	0.40	0.45

The incident solar radiation rate column gives the average annual solar radiation rate incident to the exterior southern wall surface as returned by EnergyPlus’ output variable *Surface Outside Face Incident Solar Radiation Rate per Area*.

**Fig 1:**
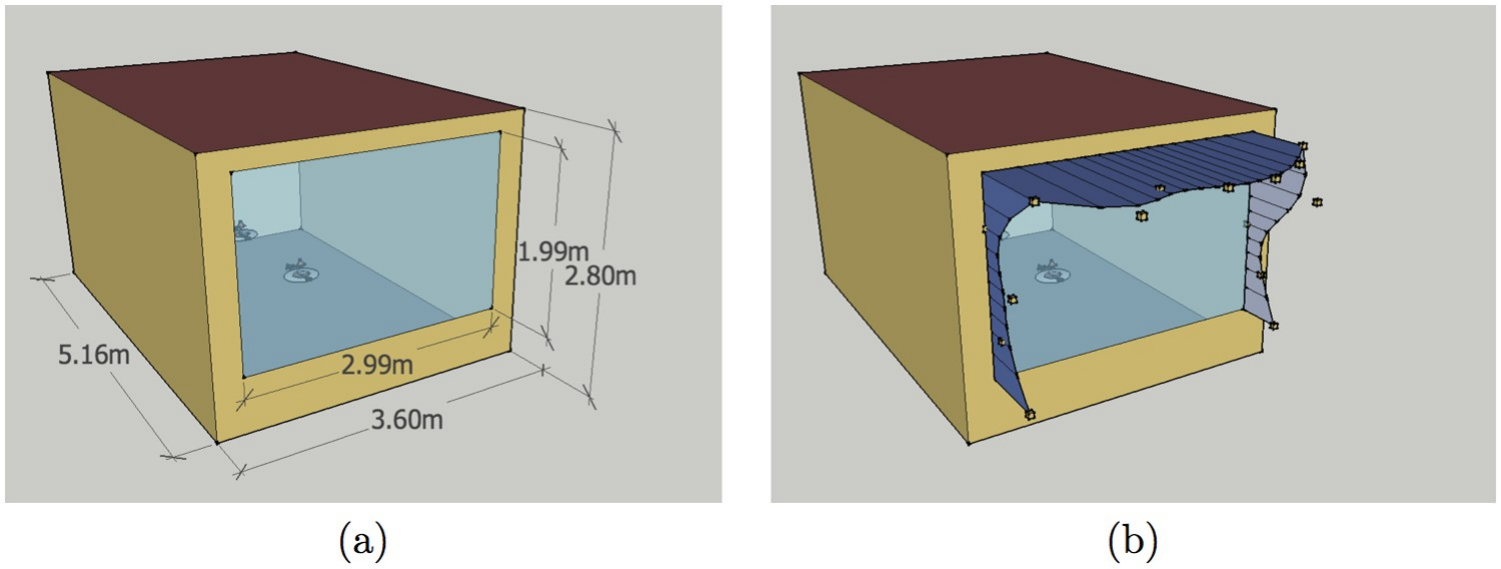
Cellular office model used in simulations: (a) dimensions; (b) small boxes indicate positions of 15 control points of NURBS curves.

The present study aims to study effects of shading a single office window, so that a cellular office has been set up as a single zone, using settings, materials, constructions and schedules of the underlying PNNL models. The office has width 3.60m, height 2.80m and depth 5.16m, due to the 200 ft^2^/person requirement from [25]. The external wall is oriented toward south, while other walls are assumed to be adiabatic. In order to keep the same windows-to-wall ratio as in the PNNL office building model, the window of the cellular office has been set to be of width 2.99m and height 1.99m, with the window sill at 0.6m from the floor. The office model is illustrated in Fig 1(a). Window glazing properties depend on climate zone, and their U-values and solar heat gain coefficients are listed in Table 1. Lighting power density is set at 1 W/ft^2^. The office has daylighting control with two sensors placed at desk level at one third and two thirds of the office depth, and illuminance setpoint of 375 lux. Since the simulations are run for a single office instead of a whole building, detailed heating, ventilation and air-conditioning (HVAC) from the PNNL models had been replaced with IdealLoadsAirSystem.

Two EnergyPlus simulation parameters are important for proper calculation of shading effects. The calculation method field of the *ShadowCalculation* object is set to *TimeStepFrequency* to perform solar path, shadowing and diffuse sky modeling calculations at each timestep (set at 15 minutes). The solar distribution field of the *Building* object is then set to *FullInteriorAndExterior* to compute shadow patterns on exterior surfaces by the window shading and to calculate amounts of transmitted beam radiation falling on each internal surface by projecting the sun’s rays through the window.

Archive with simulation files for a cellular office model for all locations is available at [28].

### NURBS curves and epnurbs package

NURBS is a widely accepted standard in computer-aided design, engineering and manufacturing for describing and generating smooth curves and surfaces [29, 30]. A NURBS curve is defined by a sequence of control points *P*_*i*_, *i* ∈ *I* for some index set *I*, that act as if *P*_*i*_ were connected to the curve by a spring of strength *w*_*i*_. Each point of the NURBS curve *C*(*t*), 0 ≤ *t* ≤ 1, is actually a convex combination of control points:

$$C\left(t\right)=\frac{\sum_{i\in I}w_{i}N_{i}\left(t\right)P_{i}}{\sum_{i\in I}w_{i}N_{i}\left(t\right)},$$

where *N*_*i*_(*t*) are suitably calculated basis functions. The basis functions are determined by a degree *d* and a knot vector which partitions the interval [0, 1] into knot spans, in such a way that ∑_*i*∈*I*_ *N*_*i*_(*t*) = 1 holds for each *t* ∈ [0, 1] and that each basis function has *d* + 1 consecutive knot spans on each of which it reduces to a polynomial of degree *d* while it is equal to zero outside these knot spans. These conditions ensure that each curve point is determined by *d* + 1 closest control points. Details of computation of basis functions may be found in [29, 30].

The window shading for the cellular office model consists of three parts: western fin, overhang and eastern fin. They are placed tightly around the window in vertical planes (fins) or in a horizontal plane (overhang), with their outer edges modeled as NURBS curves. There is a total of 15 control points:

$$\begin{array}{llll} P_{i} & =(0.3, & y_{i},0.6+0.5i) & \text{for}\;i=0,\ldots ,4\text{,}\\ P_{i} & =(0.3+0.5\left(i-4\right), & y_{i},2.6) & \;\text{for}\;i=4,\ldots ,10\text{,}\\ P_{i} & =(3.3, & y_{i},2.6-0.5\left(i-10\right)) & \;\text{for}\;i=10,\ldots ,14\text{,} \end{array}$$

whose coordinates are given with respect to the lower left corner of the outside surface of the exterior wall. The NURBS curve of the western fin is determined by control points *P*_0_, …, *P*_4_, the one of the overhang by control points *P*_4_, …, *P*_10_, and that of the eastern fin by control points *P*_10_, …, *P*_14_. All control points are of unit weight. For each curve the basis functions are of degree three and are determined by a clamped uniform knot vector, that starts and ends with three empty knot spans, which ensures that the curve starts with its first control point and ends with its last control point. Due to the joint control points *P*_4_ and *P*_10_, ends of the overhang coincide with the upper ends of fins, giving the shading shape a continuous look. It should be noted, however, that internal control points of curves do not necessarily belong to them, so that the *y*_*i*_ values do not represent actual shading depths, but only the (negative) distances of control points from the external wall. An example of window shading for the cellular office model, with control points shown as small boxes, is illustrated in Fig 1(b).

In addition to smoothness, another benefit of using NURBS to model outer edge of shading is possibility to control the size of the search space. Here it is prescribed that *y*_*i*_ takes its value from the set of alternatives {0, 0.25, 0.5, 0.75, 1, 1.25, 1.5, 1.75, 2} for each *i* = 0, …, 14, so that the search space contains a total of 9^15^ feasible curves. Although large, this set of NURBS curves with uniformly distributed control points, with clamped uniform vector and unit weights certainly cannot serve to approximate all imaginable smooth curves that could be used as outer edge of shading of depth at most 2m. Nevertheless it still provides a representative *finite* sample of the latter *infinite* set. The fact that NURBS curves are determined by a relatively small number of parameters, in this case by the *y*_*i*_ values for *i* = 0, …, 14, further makes it possible to search for the optimal shading shape by using genetic optimisation. The number of control points (15) and their feasible positions (9 for each) were chosen here so that the resulting size of the search space (9^15^ candidate curves) is, in our opinion, neither too small to give a poor sample of all possible smooth shading designs nor too big for genetic optimisation.

Before that, however, another obstacle had to be overcome as EnergyPlus can model building geometry using rectilinear surfaces (with at most four vertices) only and cannot handle NURBS curves directly. A general solution for this problem is to uniformly divide the domain interval [0, 1] by points *t*_*i*_ = *i*/*k* for *i* = 0, …, *k* and a selected positive integer *k*, and then to approximate NURBS lined shading with a number of adjacent trapezoidal shadings, where the *i*-th shading, for *i* = 0, …, *k* − 1, has as vertices the curve points *C*(*t*_*i*_), *C*(*t*_*i*+1_) and their projections on the wall surface. In preliminary studies [22, 23] calculation of NURBS curve points was hard coded in the cellular office model using the EnergyPlus macro language EPMacro. Due to the absence of programming loops in EPMacro, calculation code had to be rewritten for each point, which made this a rather cumbersome solution that is not easy to update. Coding was further complicated by the fact that EnergyPlus does not allow surfaces that have a side shorter than 0.01m. This is hard to control in advance as the domain interval [0, 1] is not mapped uniformly to a NURBS curve: parts with higher curvature require a larger portion of the domain, while a smaller portion of the domain is sufficient to represent parts with lower curvature.

In order to have a solution that is easily applicable to different models, we developed a Python3 package epnurbs that creates a NURBS lined shading for a general wall surface in EnergyPlus. Its source code is openly available at [31], while the simplest way to install it is by issuing terminal command pip install epnurbs, which also installs necessary dependencies: eppy [32, 33] to handle reading, updating and writing EnergyPlus files and NURBS-Python [34, 35] to calculate NURBS curve points.

The main method of epnurbs package is createnurbsshading with signature createnurbsshading(idd_filename, idf_filename, base_surface, shading_str, ctrl_points, evaluated_points = 20). Arguments have the following meaning:

- idd_filename is a path to the EnergyPlus idd file, which contains definitions of all objects in a particular version of EnergyPlus;
- idf_filename is a path to the EnergyPlus idf or imf file, which contains definition of the building model;
- base_surface is the name of the surface in the building model to which NURBS lined shading should be attached;
- shading_str is the string containing definition of shading objects that will be created as an approximation of NURBS lined shading. This string may contain the following placeholders that will be replaced with actual values:
  - <IDX> is replaced by the ordinal number of the shading object used in approximation;
  - <BASESURFACE> is replaced by the name of the base surface;
  - <VERTICES> is replaced by a list of vertex coordinates in counterclockwise order;
  - <COUNTERVERTICES> is replaced by a list of vertex coordinates in clockwise order.
  A sample shading string used in this study is ’Shading:Zone:Detailed, Shading<IDX>, <BASESURFACE>, , , <VERTICES>;’;
- ctrl_points is a list of coordinates of control points for the NURBS curve defining outer edge, where each control point is given as a triplet [X, Y, Z] of its coordinates calculated with respect to the zone that contains the base surface;
- evaluated_points is the number of points to be calculated on the NURBS curve, which is also equal to the number of trapezoids that will be created to approximate the NURBS shading. If not given, its default value is 20.

Method createnurbsshading first loads the EnergyPlus idf file and finds the base surface, and then calculates NURBS curve points and their feet of perpendiculars to the base surface. For each pair of consecutive curve points it then creates a trapezoidal shading object with the pair of curve points and their feet of perpendiculars as vertices and adds it to the EnergyPlus idf file, provided that all sides of such trapezoid are at least 0.01m.

Package epnurbs currently contains two more methods: createnurbsopening that approximates a NURBS lined window with a set of rectangles, and createrectshading that creates a sequence of rectangular shadings with given depths. These methods are not used in the present study, so further details about them may be found in [31].

### Simulation management with jEPlus and jEPlus+EA

EnergyPlus simulations for locations mentioned in Table 1 were managed with jEPlus [36–38]. jEPlus enables one to perform parametric EnergyPlus simulations by describing a search space with sets of alternative values for specified simulation parameters and running simulations either for the whole search space or its representative sample. Parameters in this study are the positions of the control points *P*_0_, …, *P*_14_, encoded with integers from {0, 1, …, 8} which are multiplied with -0.25m to produce the respective *y*_*i*_ coordinate. jEPlus also provides the ability to call Python methods to preprocess simulation files: it supplies the method with the names of three folders that contain project files, simulation results and EnergyPlus idd file, and other arguments specified in the parameter definition, passed in as a comma-delimitted string. Python preprocessing method then creates a list of alternative positions of control points, selects the appropriate positions based on current parameter values and calls createnurbsshading from epnurbs to add to the cellular office model the western fin approximated with 10 trapezoids, the overhang approximated with 15 trapezoids and the eastern fin approximated with 10 trapezoids, after which the model is simulated with EnergyPlus v8.4.

However, jEPlus cannot be used directly in the search for optimal shading shape due to the prohibitively large search space and instead an optimisation method has to be applied. Coupling of building energy simulation tools with optimisation methods has become mainstream in the study of energy and buildings after Caldas and Norford [39] used it prominently to facilitate performance-based façade design. A number of reviews on this topic are available: Machairas et al. [40] review methods and tools used for the building design optimisation while, more specifically, Kheiri [41] reviews optimisation methods for building geometry and envelope design and Stevanović [42] reviews work on optimisation of passive solar design of buildings. Although different optimisation methods have been used in building design optimisation, such as direct search, simulated annealing, particle swarm optimisation, harmony search or ant colony optimisation, they appear rather sporadically in literature, while large majority of building design optimisation studies rely on genetic algorithms, which tend to work well for problems considered in this area an are used in this study as well. In this aspect, one might object that building design optimisation community is somewhat lagging behind other engineering communities, in which newer optimization methods, such as gravitational search [43, 44] or Jaya [45], are more easily embraced and applied in research (see, e.g., [46–49]).

Genetic algorithms, inspired by biological evolution, work by evolving a population of candidate solutions for the optimisation problem over a number of generations by repeated application of selection, reproduction, mutation and recombination, with the goal of improving candidates’ fitness, which is given by the problem’s objective function. The most well known genetic algorithm variant is the non-dominated sorting genetic algorithm NSGA-II [50], which is implemented in the jEPlus-based optimisation tool jEPlus+EA [51] used for this study. Note that Python preprocessing in jEPlus+EA is not available in versions prior to v1.7.7 beta. For each model location, population size was set to 50, somewhat larger than the number of bits needed to describe parameter values. Crossover rate was set at 90% to quicker explore part of the search space generated by current population. Mutation rate was set at relatively high *p*_mut_ = 10%, aiming to make possible to explore wider part of the search space, but without losing good solutions since NSGA-II is an elitist strategy. The population could be expected to become mature after 3/*p*_mut_ = 30 generations [52], so that the population was set to evolve for at most 100 generations here.

For each individual variant of the cellular office model, described by the parameters (*P*_0_, …, *P*_14_), EnergyPlus reports after simulation:

- annual energy used for district heating, which is the heating load *H*(*P*_0_, …, *P*_14_),
- annual energy used for district cooling, which is the cooling load *C*(*P*_0_, …, *P*_14_), and
- annual amount of electricity used for interior lights, which is the lighting load *L*(*P*_0_, …, *P*_14_).

Since these loads use different types of end energy, they were converted to equivalent source energy. According to [53], district heating has efficiency 0.3 and uses natural gas with source energy factor 1.092, so that the heating load is multiplied by

$$c_{h}=1.092/0.3=3.64$$

to obtain equivalent source energy. District cooling has COP of 3.0 and uses electricity with source energy factor 3.317, so that the cooling load is multiplied by

$$c_{c}=3.317/3.0\approx 1.10567,$$

while the lighting load is multiplied just by the source energy factor

$$c_{l}=3.317.$$

Thus genetic algorithm was set to minimize the objective function

$$ESE\left(P_{0},\ldots ,P_{14}\right)=c_{h}H\left(P_{0},\ldots ,P_{14}\right)+c_{c}C\left(P_{0},\ldots ,P_{14}\right)+c_{l}L\left(P_{0},\ldots ,P_{14}\right),$$

which represents equivalent source energy for heating, cooling and lighting loads, under the constraint that *P*_0_, …, *P*_14_ ∈ {0, 1, …, 8}. Following this optimisation, exhaustive search was performed in a small neighborhood of the best solution found by jEPlus+EA in order to fine-tune optimal positions of control points.

## Results and discussion

### Convergence of solutions

**Fig 2:**
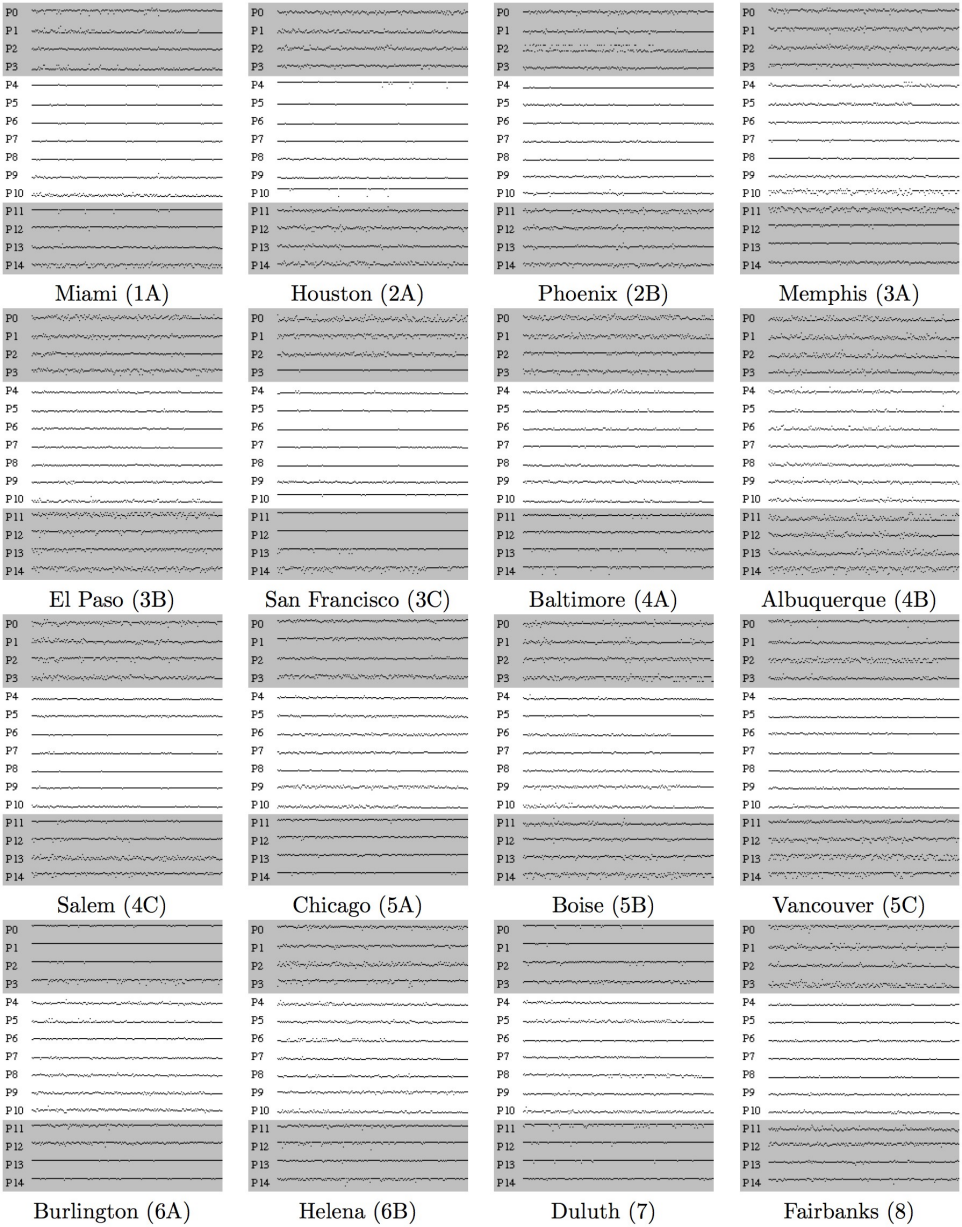
Positions of control points of the best 200 candidate solutions found by genetic optimisation for each model location. Solutions are ordered from left to right in decreasing order of total equivalent source energy, *ESE*, with positions of control points indicated in labeled rows. Position of each control point is indicated by height of the appropriate pixel, with the highest position corresponding to value 0 (×0.25m), and the lowest position corresponding to value 8 (×0.25m).

With the search space consisting of 9^15^ ≈ 2.06 × 10^14^ shading variants, the first question to tackle is whether the process of genetic optimisation had converged to a local optimum (which need not be a global optimum). Since NSGA-II keeps fittest candidates over different generations, total population created over 100 generations for each model location consisted on average of 4300 shadings that were simulated in EnergyPlus. Positions of control points of the best 200 candidates found during optimisation for each model location are shown in Fig 2.

It can be seen from these diagrams that positions of the overhang control points *P*_4_, …, *P*_10_ converge in ten model locations (Miami, Houston, Phoenix, El Paso, San Francisco, Baltimore, Salem, Boise, Vancouver and Fairbanks), but do not converge in the remaining six locations (Memphis, Albuquerque, Chicago, Burlington, Helena and Duluth). While this divergence is in most cases due to oscillation of control point positions between two adjacent values, one can see that *P*_4_ in Memphis, Albuquerque and Burlington, *P*_9_ in Albuquerque, Chicago, Burlington and Helena, and *P*_10_ in Memphis, Burlington and Duluth take on wider range of values in solutions closer to the optimal one found.

Situation is rather opposite with the fin control points *P*_0_, …, *P*_3_ and *P*_11_, …, *P*_14_: while some of them clearly converge like *P*_3_ in San Francisco, *P*_11_ in Miami or *P*_12_ in Memphis, most of them do not seem to be determined in candidates close to the optimal one. One feasible explanation for this situation is that positions of fin control points have less influence on total equivalent source energy than positions of overhang control points, which forces the genetic algorithm to decide on values of more important parameters first, and leave fine-tuning of less important parameters for future generations. It is thus possible that positions of the fin control points would converge if genetic algorithm were let to run for additional number of generations. However, one should note that running genetic optimisation in jEPlus+EA for additional 100 generations requires approximately 6-7 hours of computing time on a 4-core desktop workstation for each of 16 model locations. In addition, one cannot be certain how many additional generations are needed to achieve convergence: genetic optimisation for Albuquerque was run for additional 100 generations and while the fin control points *P*_2_ and *P*_3_ started to converge, the remaining fin control points did not achieve convergence.

**Fig 3:**
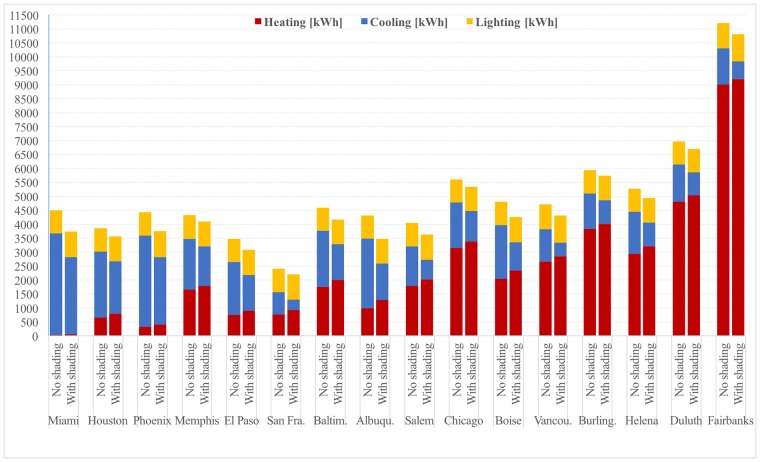
Heating, cooling and lighting loads in models without shading and with optimal shading.

**Fig 4:**
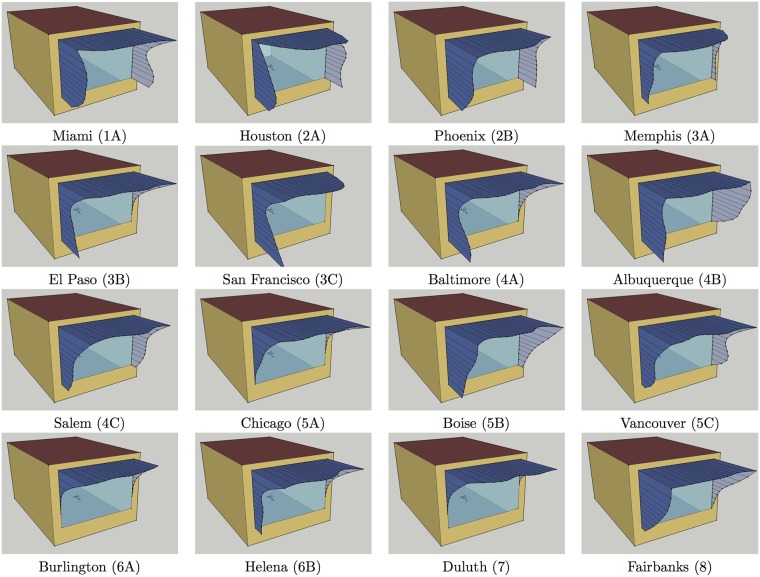
Sketchup visualisations of cellular office model with optimal shading found for each location.

Convergence of positions of control points can also be observed through decreasing standard deviations of positions in best candidate solutions: Table 2 shows standard deviations of positions for the best 200, the best 100 and the best 50 candidates for each model location, together with the average values of positions of control points in the best 50 candidates. Based on information in this table and diagrams in Fig 2, for each model location it was possible to select positions of control points that have converged and to select 2-3 most occurring positions for the remaining control points (except for *P*_12_ in Houston and *P*_13_ in Vancouver, where four alternatives were selected in each case). These selections, shown in Table 3, determine neighborhoods of the best solutions found by genetic optimisation which consist of 64–576 shading variants. An exhaustive search was additionally performed with jEPlus in these neighborhoods, which led to subtle improvements in optimal shading for all model locations other than Vancouver. Table 4 shows positions of control points of the optimal shading variants, together with heating, cooling and lighting loads in equivalent source energy terms for both the model with the optimal shading and the starting model without shading. For easier comparison, these loads are visually represented in Fig 3 as well. Fig 4 futher shows a Sketchup visualisation of the model with optimal shading found for each location.

**Table 2:** Standard deviations of positions of control points in best shadings found by genetic optimisation for each model location.

Location		*P*_0_	*P*_1_	*P*_2_	*P*_3_	*P*_4_	*P*_5_	*P*_6_	*P*_7_	*P*_8_	*P*_9_	*P*_10_	*P*_11_	*P*_12_	*P*_13_	*P*_14_
Miami	*σ*_200_	1.09	1.19	0.65	1.15	0.22	0.21	0.23	0.26	0.23	0.59	0.90	0.53	0.57	0.64	1.44
	*σ*_100_	0.99	0.85	0.61	0.78	0.26	0.17	0.24	0.26	0.22	0.64	0.79	0.30	0.35	0.58	1.42
	*σ*_50_	0.53	0.78	0.53	0.67	0.20	0.20	0.24	0.30	0.24	0.46	0.56	0.00	0.27	0.53	1.44
	*av*_50_	2.72	6.70	5.28	7.52	5.04	6.96	7.06	7.10	7.06	7.22	7.62	3.00	2.08	4.86	4.58
Houston	*σ*_200_	1.03	1.05	1.09	0.87	0.74	0.12	0.14	0.17	0.45	0.43	0.99	0.84	1.16	0.78	1.38
	*σ*_100_	1.07	0.88	1.08	0.86	1.02	0.10	0.10	0.14	0.37	0.33	0.80	0.70	0.90	0.67	1.23
	*σ*_50_	1.15	0.80	0.91	0.97	0.84	0.00	0.00	0.00	0.27	0.24	0.00	0.40	0.90	0.47	1.20
	*av*_50_	4.46	6.42	4.74	3.98	2.12	6.00	8.00	7.00	6.92	7.94	0.00	3.96	3.28	3.76	3.40
Phoenix	*σ*_200_	0.96	0.77	2.59	0.67	0.12	0.35	0.36	0.44	0.25	0.51	0.63	1.09	0.85	0.78	1.15
	*σ*_100_	0.96	0.57	2.17	0.57	0.00	0.22	0.38	0.43	0.20	0.45	0.50	1.30	0.73	0.80	1.11
	*σ*_50_	0.91	0.34	0.75	0.38	0.00	0.20	0.41	0.24	0.00	0.24	0.42	1.29	0.61	0.66	0.99
	*av*_50_	3.98	5.96	7.56	6.12	8.00	6.96	7.22	7.94	8.00	5.98	4.94	4.38	3.22	3.80	4.24
Memphis	*σ*_200_	1.00	1.19	1.21	0.90	0.95	0.71	0.58	0.41	0.33	0.60	1.63	1.60	0.56	0.35	0.81
	*σ*_100_	0.99	1.16	1.19	0.75	1.01	0.70	0.56	0.43	0.34	0.55	1.67	1.57	0.50	0.38	0.77
	*σ*_50_	0.74	1.02	1.07	0.63	0.89	0.38	0.47	0.45	0.20	0.51	1.45	1.73	0.20	0.30	0.59
	*av*_50_	2.64	2.36	4.26	2.62	5.66	6.82	6.66	6.28	6.00	6.02	2.82	2.90	0.04	0.10	1.64
El Paso	*σ*_200_	1.38	1.10	0.82	1.45	0.56	0.54	0.47	0.52	0.45	0.55	0.94	1.56	1.11	1.15	1.48
	*σ*_100_	1.30	0.91	0.59	1.57	0.52	0.52	0.37	0.30	0.43	0.52	0.87	1.62	0.84	1.13	1.45
	*σ*_50_	1.27	0.70	0.54	1.53	0.45	0.37	0.20	0.00	0.40	0.49	0.88	1.72	0.83	1.27	1.52
	*av*_50_	4.22	4.52	4.10	2.30	6.80	7.84	7.00	8.00	7.20	6.04	7.54	2.62	0.46	0.86	2.24
San Francisco	*σ*_200_	1.77	1.37	1.11	0.23	0.57	0.23	0.12	0.38	0.12	0.53	0.24	0.18	0.07	0.86	1.65
	*σ*_100_	1.85	1.45	1.11	0.22	0.55	0.22	0.10	0.45	0.10	0.47	0.24	0.10	0.00	0.37	1.59
	*σ*_50_	2.05	1.40	0.99	0.20	0.37	0.20	0.00	0.49	0.00	0.42	0.27	0.14	0.00	0.24	1.10
	*av*_50_	6.02	3.86	5.02	2.04	7.84	7.00	8.00	7.42	8.00	6.16	0.08	0.02	0.00	0.06	1.06
Baltimore	*σ*_200_	1.57	1.52	0.72	1.15	0.78	0.52	0.56	0.35	0.50	0.65	0.64	0.72	0.59	0.69	1.12
	*σ*_100_	1.51	1.53	0.70	1.01	0.62	0.50	0.48	0.31	0.43	0.63	0.54	0.78	0.60	0.75	0.54
	*σ*_50_	1.51	1.59	0.64	0.82	0.28	0.32	0.40	0.20	0.27	0.52	0.35	0.65	0.63	0.84	0.43
	*av*_50_	4.52	5.26	3.10	3.34	6.96	7.88	7.86	6.04	7.92	5.74	7.86	2.68	1.28	0.34	0.18
Albuquerque	*σ*_200_	1.50	1.52	1.53	1.13	0.87	0.71	1.02	0.56	0.75	0.90	0.86	2.00	1.32	1.59	2.04
	*σ*_100_	1.43	1.49	1.41	1.02	0.62	0.83	0.75	0.50	0.55	0.74	0.84	2.24	1.20	1.67	2.04
	*σ*_50_	1.20	1.53	1.18	0.81	0.54	0.88	0.35	0.31	0.42	0.71	0.72	2.56	0.78	1.64	1.78
	*av*_50_	6.38	6.34	7.14	4.94	6.06	7.78	7.86	6.06	7.16	6.18	6.28	6.28	5.10	5.12	1.88
Salem	*σ*_200_	1.30	1.18	1.24	1.28	0.49	0.53	0.17	0.44	0.12	0.29	0.46	0.44	0.78	1.41	1.00
	*σ*_100_	1.17	0.98	1.05	1.07	0.46	0.56	0.10	0.35	0.00	0.17	0.29	0.31	0.70	1.36	0.86
	*σ*_50_	1.16	0.70	0.82	1.01	0.43	0.50	0.14	0.31	0.00	0.14	0.31	0.20	0.57	1.27	0.65
	*av*_50_	2.94	4.42	2.62	4.24	7.76	6.50	7.02	7.94	8.00	6.02	6.94	3.00	2.60	3.98	1.02
Chicago	*σ*_200_	0.75	0.73	0.63	1.30	0.57	0.68	0.73	0.56	0.39	1.01	0.83	0.56	0.62	0.55	0.38
	*σ*_100_	0.71	0.71	0.59	1.23	0.51	0.70	0.77	0.46	0.40	0.98	0.67	0.57	0.49	0.46	0.31
	*σ*_50_	0.72	0.73	0.63	1.08	0.50	0.72	0.75	0.40	0.44	0.90	0.37	0.24	0.45	0.30	0.39
	*av*_50_	1.00	0.84	2.72	3.60	6.30	6.72	6.80	6.80	7.26	5.56	7.84	1.06	0.28	0.10	0.08
Boise	*σ*_200_	1.13	1.13	1.12	1.63	0.65	0.28	0.65	0.39	0.62	0.85	1.01	0.99	0.71	1.06	1.74
	*σ*_100_	1.04	1.14	1.17	1.90	0.52	0.14	0.52	0.30	0.57	0.86	0.60	0.82	0.60	1.18	1.77
	*σ*_50_	1.01	0.98	1.15	2.02	0.49	0.20	0.41	0.14	0.40	0.85	0.45	0.45	0.58	0.85	1.74
	*av*_50_	4.16	4.80	3.50	4.32	7.60	6.00	7.90	7.02	7.80	4.86	7.80	5.96	3.02	1.90	2.18
Vancouver	*σ*_200_	0.96	0.83	1.68	0.93	0.51	0.40	0.48	0.35	0.58	0.50	0.63	0.85	1.40	1.99	1.60
	*σ*_100_	0.90	0.80	1.72	0.77	0.49	0.30	0.35	0.24	0.57	0.35	0.45	0.87	1.35	2.15	1.32
	*σ*_50_	0.61	0.82	1.64	0.86	0.40	0.20	0.20	0.00	0.45	0.00	0.38	0.72	1.02	2.27	0.95
	*av*_50_	1.50	5.08	4.12	4.78	7.80	7.96	6.00	8.00	7.80	7.00	7.82	3.08	2.36	3.36	0.64
Burlington	*σ*_200_	0.37	0.00	0.27	1.24	0.78	0.76	0.40	0.55	0.68	0.76	0.89	0.77	0.82	0.16	0.32
	*σ*_100_	0.34	0.00	0.14	1.35	0.77	0.45	0.39	0.52	0.57	0.76	0.80	0.48	0.68	0.14	0.27
	*σ*_50_	0.30	0.00	0.00	1.46	0.66	0.49	0.45	0.50	0.50	0.72	0.83	0.43	0.54	0.00	0.27
	*av*_50_	0.10	0.00	0.00	0.80	6.28	6.80	5.14	6.56	5.84	5.72	4.46	1.12	0.52	0.00	0.08
Helena	*σ*_200_	0.94	0.81	1.44	1.24	0.88	0.71	0.90	0.57	0.62	0.76	0.83	0.77	1.03	0.51	1.02
	*σ*_100_	0.95	0.65	1.26	1.26	0.67	0.72	0.58	0.48	0.61	0.84	0.69	0.57	0.48	0.43	0.83
	*σ*_50_	1.01	0.69	1.16	0.70	0.67	0.54	0.43	0.43	0.37	0.79	0.58	0.38	0.40	0.35	0.57
	*av*_50_	1.98	2.60	3.12	1.16	7.54	6.84	7.76	7.76	6.94	4.36	6.68	1.82	0.14	0.14	0.28
Duluth	*σ*_200_	0.50	0.25	0.81	0.83	0.65	0.89	0.36	0.46	1.10	0.59	0.87	1.13	0.50	0.46	0.47
	*σ*_100_	0.43	0.00	0.84	0.80	0.40	0.89	0.32	0.32	1.23	0.50	0.77	1.10	0.24	0.33	0.46
	*σ*_50_	0.00	0.00	0.62	0.85	0.32	0.82	0.24	0.14	1.23	0.43	0.76	1.10	0.14	0.20	0.48
	*av*_50_	0.00	0.00	0.18	2.20	5.88	6.60	7.06	5.98	6.58	6.24	6.24	0.48	0.02	0.04	0.26
Fairbanks	*σ*_200_	1.09	1.33	1.01	1.86	0.44	0.39	0.52	0.50	0.32	0.54	0.69	1.13	1.02	0.61	0.73
	*σ*_100_	0.90	1.09	0.99	1.55	0.43	0.41	0.52	0.47	0.26	0.44	0.68	1.03	0.89	0.54	0.79
	*σ*_50_	0.93	1.15	0.71	1.36	0.41	0.40	0.50	0.30	0.20	0.36	0.60	0.81	0.88	0.49	0.69
	*av*_50_	1.12	4.00	4.98	7.10	7.78	7.14	7.56	7.90	7.96	6.90	7.04	5.78	1.54	1.80	1.04

Standard deviations of positions of control points for the best 200 (*σ*_200_), the best 100 (*σ*_100_) and the best 50 (*σ*_50_) shadings for each model location, together with average values of positions of control points for the best 50 (*av*_50_) shadings found by genetic optimisation.

**Table 3:** Neighborhoods used in local search to improve the best solution found by genetic algorithm.

Location	*P*_0_	*P*_1_	*P*_2_	*P*_3_	*P*_4_	*P*_5_	*P*_6_	*P*_7_	*P*_8_	*P*_9_	*P*_10_	*P*_11_	*P*_12_	*P*_13_	*P*_14_
Miami	2, 3	**7**	5, 6	6, 7, 8	**5**	**7**	**7**	**7**	**7**	7, 8	6, 7, 8	**3**	**2**	4, 5, 6	**4**
Houston	**4**	**7**	3, 5	3, 4, 5	**2**	**6**	**8**	**7**	**7**	**8**	**0**	3, 4, 5	2, 3, 4, 5	3, 4	**4**
Phoenix	**4**	**6**	**8**	6, 7	**8**	**7**	7, 8	**8**	**8**	5, 6	4, 5, 6	3, 4, 5	3, 4	3, 4	**4**
Memphis	**2**	**2**	**5**	2, 3	5, 6, 7	6, 7	6, 7	6, 7	**6**	6, 7	2, 5	**2**	**0**	**1**	**1**
El Paso	**5**	4, 5	**4**	1, 2	6, 7	7, 8	**7**	**8**	7, 8	5, 6, 7	7, 8	**3**	**0**	**0**	**0**
San Francisco	2, 8	3, 6	**5**	**2**	7, 8	**7**	**8**	7, 8	**8**	6, 7	0, 1	**0**	**0**	0, 1	0, 1
Baltimore	**6**	**7**	**3**	**3**	6, 7, 8	7, 8	7, 8	6, 7	7, 8	5, 6	7, 8	**3**	**1**	**0**	**0**
Albuquerque	**6**	**6**	**8**	**5**	5, 6, 7	7, 8	7, 8	6, 7	7, 8	5, 6, 8	6, 7	3, 8	**5**	**6**	**0**
Salem	**2**	**4**	2, 3	3, 4, 5	7, 8	6, 7	**7**	7, 8	**8**	**6**	6, 7	**3**	2, 3	3, 5	**1**
Chicago	**0**	**0**	**3**	3, 5	6, 7	6, 7, 8	6, 7	6, 7	7, 8	6, 7	7, 8	**1**	**0**	**0**	**0**
Boise	**4**	**5**	3, 5	3, 8	7, 8	**6**	**8**	**7**	7, 8	4, 5	7, 8	**6**	**3**	1, 2	1, 5
Vancouver	**1**	**5**	**3**	**5**	7, 8	**8**	**6**	**8**	7, 8	**7**	7, 8	**3**	**2**	0, 1, 5, 6	0, 1
Burlington	**0**	**0**	**0**	0, 1	5, 6, 7	6, 7	5, 6	6, 7	5, 6	**6**	4, 5	**1**	0, 1	**0**	**0**
Helena	**2**	**2**	3, 4	**1**	7, 8	6, 7	7, 8	7, 8	6, 7	4, 5	6, 7	**2**	**0**	**0**	**0**
Duluth	**0**	**0**	**0**	**2**	5, 6	6, 7	7, 8	**6**	6, 8	6, 7	5, 6, 7	**0**	**0**	**0**	**0**
Fairbanks	**1**	**4**	5, 6	**8**	7, 8	7, 8	7, 8	7, 8	**8**	6, 7	6, 7, 8	**6**	**1**	**1**	**0**

Control points whose positions appear to have converged during genetic optimisation have just one value listed above in bold, while the most occurring values among best candidate solutions are listed for the remaining control points.

**Table 4:** Positions of control points in optimal shading found for each location.

Location	Without shading				Positions of control points	With optimal shading			
	*c*_*h*_*H*_none_	*c*_*c*_*C*_none_	*c*_*l*_*L*_none_	*ESE*_none_		*c*_*h*_*H*_opt_	*c*_*c*_*C*_opt_	*c*_*l*_*L*_opt_	*ESE*_opt_
Miami	30.4	3637.1	833.0	4500.5	(3, 7, 6, 8, 5, 7, 7, 7, 7, 7, 8, 3, 2, 6, 4)	56.8	2762.1	924.4	3743.3
Houston	656.2	2369.8	840.4	3866.4	(4, 7, 5, 4, 2, 6, 8, 7, 7, 8, 0, 5, 4, 3, 4)	793.1	1881.8	891.6	3566.5
Phoenix	319.4	3274.9	838.9	4433.2	(4, 6, 8, 6, 8, 7, 8, 8, 8, 6, 6, 3, 4, 4, 4)	400.4	2414.7	933.4	3748.5
Memphis	1654.7	1825.4	852.0	4332.1	(2, 2, 5, 3, 6, 7, 7, 7, 6, 6, 2, 2, 0, 1, 1)	1786.3	1416.9	903.8	4107.0
El Paso	750.8	1889.4	839.4	3479.5	(5, 4, 4, 2, 7, 8, 7, 8, 8, 7, 8, 3, 0, 0, 0)	897.3	1288.0	904.3	3089.5
San Francisco	769.4	791.2	850.8	2411.4	(8, 6, 5, 2, 8, 7, 8, 8, 8, 7, 0, 0, 0, 0, 0)	931.7	371.1	905.8	2208.6
Baltimore	1748.0	2018.4	828.2	4594.7	(6, 7, 3, 3, 7, 8, 8, 7, 8, 6, 8, 3, 1, 0, 0)	2000.2	1290.6	874.2	4165.0
Albuquerque	986.6	2506.3	820.7	4313.6	(6, 6, 8, 5, 7, 8, 8, 7, 8, 8, 7, 8, 5, 6, 0)	1286.7	1299.9	885.3	3471.8
Salem	1786.7	1415.5	845.4	4047.6	(2, 4, 3, 5, 8, 7, 7, 8, 8, 6, 7, 3, 3, 3, 1)	2013.4	714.0	904.7	3632.2
Chicago	3157.7	1619.2	835.8	5612.8	(0, 0, 3, 3, 7, 8, 7, 7, 8, 6, 8, 1, 0, 0, 0)	3380.6	1089.4	873.2	5343.2
Boise	2041.7	1927.8	842.6	4812.0	(4, 5, 5, 8, 8, 6, 8, 7, 8, 5, 8, 6, 3, 2, 1)	2335.5	1012.0	908.1	4255.6
Vancouver	2657.0	1159.8	901.1	4717.9	(1, 5, 3, 5, 8, 8, 6, 8, 8, 7, 8, 3, 2, 5, 0)	2848.9	488.3	981.0	4318.3
Burlington	3830.7	1265.9	843.1	5939.7	(0, 0, 0, 1, 7, 7, 6, 7, 6, 6, 5, 1, 0, 0, 0)	4003.8	855.6	875.9	5735.3
Helena	2943.7	1499.0	838.7	5281.3	(2, 2, 4, 1, 8, 7, 8, 8, 7, 5, 7, 2, 0, 0, 0)	3211.9	848.0	876.8	4936.7
Duluth	4806.3	1335.7	822.6	6964.7	(0, 0, 0, 2, 6, 7, 8, 6, 8, 7, 7, 0, 0, 0, 0)	5039.6	815.6	848.2	6703.3
Fairbanks	9016.8	1283.1	916.0	11215.9	(1, 4, 6, 8, 8, 8, 8, 8, 8, 7, 8, 6, 1, 1, 0)	9198.8	644.3	973.5	10816.6

*H*_none_, *C*_none_ and *L*_none_ are loads for a cellular office model without shading, while *H*_opt_, *C*_opt_ and *L*_opt_ are loads for a model with the optimal NURBS shading found after exhaustive search in the vicinity of the best solution returned by genetic optimisation. All loads are given in equivalent source energy terms, measured in kWh.

### Load changes from optimal shading

As it can be seen from Table 4, presence of shading increases heating loads in all model locations, from 26.1kWh in Miami up to 300.1kWh in Albuquerque in absolute terms. Apart from Miami, which has negligible heating load, this difference represents relative increase of between 2.0% in Fairbanks and 30.4% in Albuquerque compared to heating load of the unshaded model. Equally expected, presence of shading increases lighting loads in all model locations as well. As lighting loads are more uniformly distributed among different locations, ranging from 820.7kWh in Albuquerque to 916.0kWh in Fairbanks, relative increase of lighting load is also more uniformly distributed, ranging from 3.1% in Duluth to 11.3% in Phoenix. Increases in heating and lighting loads are, however, well compensated by decreases in cooling loads which range from 408.4kWh in Memphis to 1206.4kWh in Albuquerque in absolute terms and represent relative decrease of between 20.6% in Houston and 57.9% in Vancouver compared to cooling loads of unshaded models. When all these loads are taken together, the benefits of shading are still clear: total equivalent source energy is reduced between 202.7kWh in San Francisco and 841.7kWh in Albuquerque in absolute terms, which represents relative decrease of between 3.4% in Burlington and 19.5% in Albuquerque.

One should take into account that optimal shadings found here have overhang depths mostly between 1.5m and 2m, which renders them impractical in locations with large annual snowfall such as Chicago, Burlington, Helena, Duluth and Fairbanks. The results reported here are given for completeness of comparisons, while in practice positive effects of shading on total equivalent source energy for these locations would better be achieved by improving performance of glazing in PNNL office building models.

### Some unexpected optimal positions of control points

As genetic algorithm is allowed to freely and independently changes values of genes during the process of optimisation, informed only by changes in objective function that determine fitness of candidates, it may easily end up with optimal solutions whose characteristics appear counterintuitive at first. There are a few such unexpected phenomena in optimal shadings visualized in Fig 4: a hole in the upper western part of shading for Miami, holes in the upper western and eastern parts of shading for Houston, and protrusion of the lower part of western fin for El Paso, San Francisco and Baltimore.

**Fig 5:**
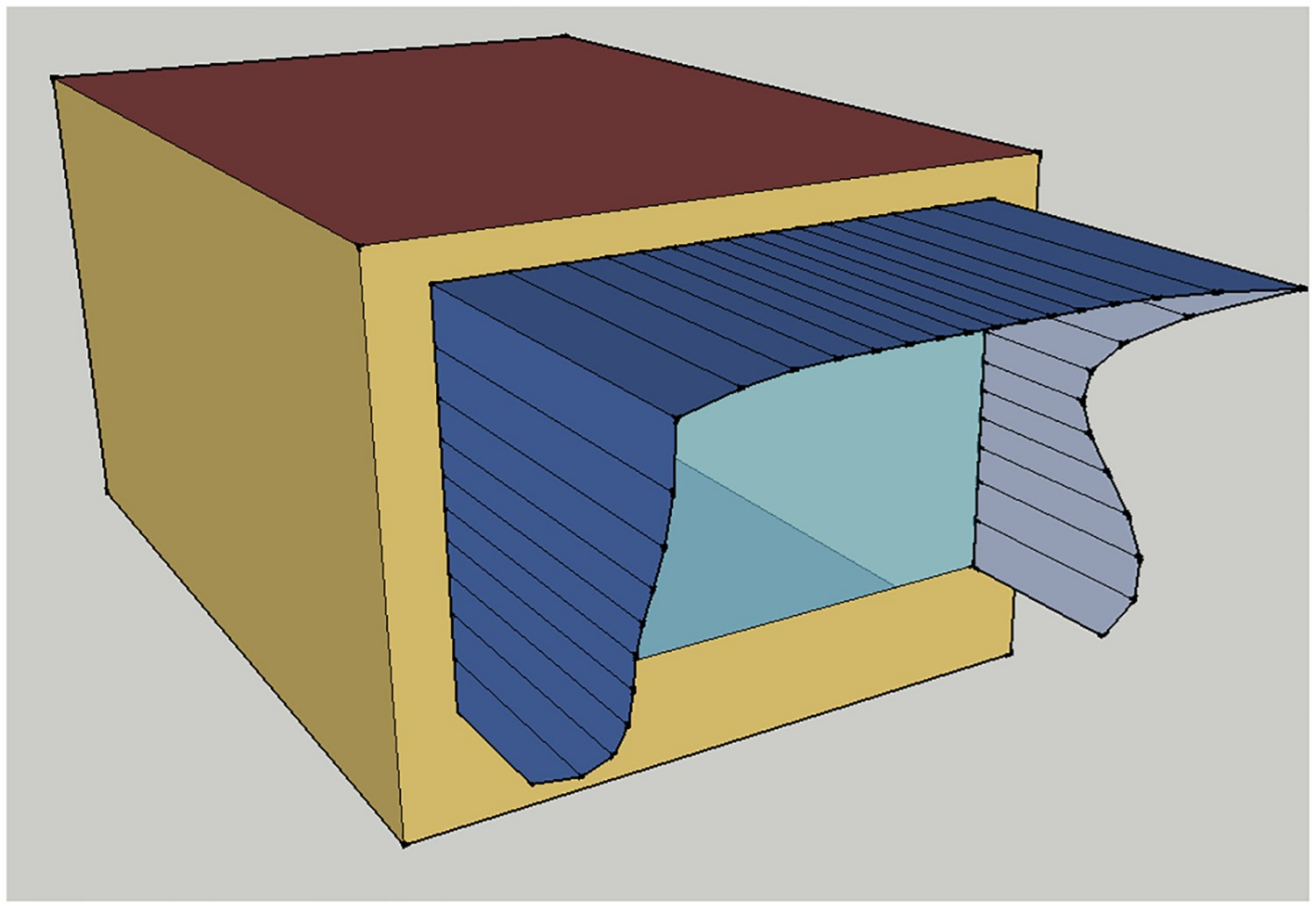
A better performing shading without a western hole in Miami with positions of control points (3, 7, 6, 8, 8, 7, 7, 7, 7, 7, 8, 3, 2, 6, 4).

Western hole in the optimal shading for Miami is clearly related to the position of control point *P*_4_, which serves as an ending point for both the western fin and the overhang. Table 5 shows heating, cooling and lighting loads of the cellular office model in Miami when the position of *P*_4_ is varied from 0 to 8, while the positions of the remaining control points in this shading are kept intact. As expected, heating and lighting loads increase, while cooling load decreases with an increase in *P*_4_, since an increase in the value of *P*_4_ also increases the shading area. However, one can see that these loads do not depend linearly on the value of *P*_4_. The minimal total equivalent source energy is obtained for the maximum value of *P*_4_, for which the shading loses its hole as shown in Fig 5. Thus, this counterintuitive hole in the optimal shading served to help improve the results of genetic optimisation, whose best candidate solutions clearly had the value of *P*_4_ wrongly converging to 5.

**Table 5:** Heating, cooling and lighting loads for cellular office model in Miami when the position of control point *P*_4_ is varied in the optimal shading.

*P*_4_	*c*_*h*_*H*	*c*_*c*_*C*	*c*_*l*_*L*	*ESE*
0	54.5	2805.8	904.4	3764.8
1	55.2	2795.9	907.8	3758.9
2	55.7	2788.4	916.9	3760.9
3	56.1	2780.0	922.2	3758.3
4	56.5	2770.5	922.3	3749.3
5	56.8	2762.1	924.4	3743.3
6	57.0	2754.6	927.2	3738.7
7	57.1	2748.0	930.1	3735.2
8	57.2	2742.6	933.9	3733.8

The remaining control points (*P*_0_, …, *P*_3_, *P*_5_, …, *P*_14_) have positions (3, 7, 6, 8, 7, 7, 7, 7, 7, 8, 3, 2, 6, 4). Loads are given in equivalent source energy terms, measured in kWh.

Two holes in the optimal shading for Houston are related to positions of the control points *P*_4_ and *P*_10_, which serve as joint points between the overhang and the fins. Table 6 shows total equivalent source energy for heating, cooling and lighting loads for cellular office model in Houston when positions of these control points are varied from 0 to 8, while positions of the remaining control points are kept intact. As in the case of Miami, when one of these control points is increased while the other one is kept fixed, the shading area is increased so that heating and lighting loads increase, while cooling loads decreases. However, total equivalent source energy *ESE* as their weighted sum behaves somewhat erratically with respect to individual changes in *P*_4_ and *P*_10_: for example, for any fixed value of *P*_4_ in Table 6 holds

$$ESE_{P_{10}=5}<ESE_{P_{10}=6}>ESE_{P_{10}=7}<ESE_{P_{10}=8},$$

while on the other hand, for any fixed value of *P*_10_ holds

$$ESE_{P_{4}=1}>ESE_{P_{4}=2}<ESE_{P_{4}=3}<ESE_{P_{4}=4}>ESE_{P_{4}=5}.$$

The minimal total equivalent source energy is obtained for *P*_4_ = 2 and *P*_10_ = 1, which contrary to the case of Miami, shows that genetic optimisation got the value of *P*_4_ correct and made just a minor mistake with *P*_10_.

**Table 6:** Total equivalent source energy for heating, cooling and lighting loads in Houston for varying positions of control points *P*_4_ and *P*_10_ in the optimal shading.

*P*_4_ \ *P*_10_	0	1	2	3	4	5	6	7	8
0	3572.3	3571.8	3579.5	3584.5	3581.2	3582.7	3585.9	3583.4	3589.4
1	3571.0	3570.8	3578.5	3584.6	3581.4	3583.0	3586.2	3583.8	3590.2
2	3566.5	3566.3	3574.1	3580.2	3577.0	3578.6	3581.9	3579.6	3585.9
3	3573.5	3574.3	3583.4	3590.2	3587.2	3589.5	3594.1	3591.8	3600.3
4	3579.9	3580.3	3590.9	3600.2	3597.2	3599.9	3604.5	3602.3	3612.3
5	3577.3	3577.8	3589.3	3598.4	3595.3	3598.1	3602.9	3600.7	3611.2
6	3575.5	3576.0	3588.3	3597.4	3594.4	3597.3	3602.1	3600.0	3610.7
7	3571.8	3572.3	3584.8	3593.8	3590.9	3593.8	3598.7	3596.6	3607.3
8	3568.5	3569.1	3581.5	3590.7	3587.7	3590.7	3595.6	3593.5	3604.3

The remaining control points (*P*_0_, …, *P*_3_, *P*_5_, …, *P*_9_, *P*_11_, …, *P*_14_) have values (4, 7, 5, 4, 6, 8, 7, 7, 8, 5, 4, 3, 4). Total equivalent source energy is measured in kWh.

Protrusions of the lower part of western fin in the optimal shadings for El Paso, San Francisco and Baltimore are consequences of a large value of the control point *P*_0_, which serves as the lower end of the western fin, compared to values of its remaining control points. Table 7 shows total equivalent source energy for heating, cooling and lighting loads for the cellular office model in these locations when the position of *P*_0_ is varied from 0 to 8 while the remaining control points in optimal shadings are kept intact. Interestingly, the minimal total equivalent source energy is obtained for *P*_0_ = 8 for El Paso which causes even larger protrusion of the western fin. The optimal value *P*_0_ = 8 for San Francisco has already been found by genetic optimisation, while the fin protrusion is slightly reduced only for Baltimore for which the optimal value is *P*_0_ = 4. However, one should note that differences in total equivalent source energy among shadings considered in Table 7 are less than 0.32%, which opens up an option to obtain shadings of improved design with only minor sacrifices in energy use.

**Table 7:** Total equivalent source energy for heating, cooling and lighting loads for cellular office model in El Paso, San Francisco and Baltimore for varying positions of the control point *P*_0_ in optimal shadings.

*P*_0_	El Paso	San Francisco	Baltimore
0	3098.3	2211.9	4167.1
1	3094.8	2210.1	4165.5
2	3091.8	2208.8	4165.1
3	3090.5	2209.4	4165.0
4	3088.9	2209.1	4164.7
5	3089.5	2209.4	4165.0
6	3089.9	2209.0	4165.0
7	3089.1	2208.8	4165.0
8	3088.6	2208.6	4165.1

Total equivalent source energy is given above in kWh. The remaining positions of control points (*P*_1_, …, *P*_14_) are equal to (4, 4, 2, 7, 8, 7, 8, 8, 7, 8, 3, 0, 0, 0) in El Paso, (6, 5, 2, 8, 7, 8, 8, 8, 7, 0, 0, 0, 0, 0) in San Francisco and (7, 3, 3, 7, 8, 8, 7, 8, 6, 8, 3, 1, 0, 0) in Baltimore.

### Simpler near-optimal shading designs

Aims for symmetry and simplified lines are natural in building design. Although the present study defined the outer edge of the overhang using seven control points *P*_4_, …, *P*_10_, and thus gave genetic algorithm the freedom to explore very different shading designs, the resulting optimal shadings turn out to have very similar positions of control points *P*_5_, …, *P*_9_ that define interior part of the overhang, suggesting that genetic algorithm may have tried to equate values of these control points altogether. Actually, the ending overhang control points *P*_4_ and *P*_10_ also have positions close to the average overhang position in all cases other than *P*_4_ in Houston and *P*_10_ in Houston, Memphis and San Francisco. This observation called for simulating additional rectifying shading modifications for each model location where:

1. positions of interior overhang control points *P*_5_, …, *P*_9_ are replaced by their average position in the optimal shading;
2. positions of western tip and interior overhang control points *P*_4_, …, *P*_9_ are replaced by their average position in the optimal shading;
3. positions of eastern tip and interior overhang control points *P*_5_, …, *P*_10_ are replaced by their average position in the optimal shading;
4. positions of all overhang control points *P*_4_, …, *P*_10_ are replaced by their average position in the optimal shading,

while positions of the remaining control points were kept intact. Table 8 gives the minimum total equivalent source energy for heating, cooling and lighting loads among these shading modification. When compared to optimal shadings from Table 4 (and their improvements for Miami, Houston, El Paso and Baltimore based on Tables 5, 6 and 7), it can be seen that rectification of the overhang increased total equivalent source by at most 0.23% (in Houston), and even slightly decreased it in 7 of 16 locations (Miami, Phoenix, San Francisco, Albuquerque, Vancouver, Duluth and Fairbanks). This gives some support to the expectation that truly optimal shadings have rectified overhang.

**Table 8:** Overhang rectification in optimal shadings.

Location	Positions of control points	*c*_*h*_*H*	*c*_*c*_*C*	*c*_*l*_*L*	*ESE*	Change (%)
Miami	(3, 7, 6, 8, 7.17 = ⋯ = 7.17, 8, 3, 2, 6, 4)	57.2	2741.7	933.9	3732.8	-0.0259
Houston	(4, 7, 5, 4, 2, 7.20 = ⋯ = 7.20, 1, 5, 4, 3, 4)	807.6	1848.2	918.9	3574.7	0.2360
Phoenix	(4, 6, 8, 6, 8, 7.40 = ⋯ = 7.40, 6, 3, 4, 4, 4)	400.3	2417.1	928.8	3746.2	-0.0594
Memphis	(2, 2, 5, 3, 6, 6.60 = ⋯ = 6.60, 2, 2, 0, 1, 1)	1784.6	1418.9	908.9	4112.4	0.1316
El Paso	(8, 4, 4, 2, 7, 7.67 = ⋯ = 7.67, 3, 0, 0, 0, 0)	899.2	1284.0	905.6	3088.8	0.0066
San Francisco	(8, 6, 5, 2, 7.67 = ⋯ = 7.67, 0, 0, 0, 0, 0)	932.1	372.1	902.9	2207.0	-0.0717
Baltimore	(4, 7, 3, 3, 7, 7.40 = ⋯ = 7.40, 8, 3, 1, 0, 0)	1995.2	1296.8	873.4	4165.4	0.0166
Albuquerque	(6, 6, 8, 5, 7.67 = ⋯ = 7.67, 7, 8, 5, 6, 0)	1288.2	1296.1	885.3	3469.6	-0.0653
Salem	(2, 4, 3, 5, 7.33 = ⋯ = 7.33, 7, 3, 3, 3, 1)	2011.6	716.8	904.6	3633.0	0.0237
Chicago	(0, 0, 3, 3, 7, 7.33 = ⋯ = 7.33, 1, 0, 0, 0)	3379.3	1090.2	874.4	5344.0	0.0139
Boise	(4, 5, 5, 8, 7.14 = ⋯ = 7.14, 6, 3, 2, 1)	2331.4	1015.9	911.3	4258.6	0.0701
Vancouver	(1, 5, 3, 5, 8, 7.40 = ⋯ = 7.40, 8, 3, 2, 5, 0)	2850.8	488.1	977.6	4316.4	-0.0436
Burlington	(0, 0, 0, 1, 6.50 = ⋯ = 6.50, 5, 1, 0, 0, 0)	4003.0	856.7	875.9	5735.6	0.0037
Helena	(2, 2, 4, 1, 7.17 = ⋯ = 7.17, 7, 0, 0, 0, 0)	3209.7	852.6	875.7	4938.0	0.0268
Duluth	(0, 0, 0, 2, 6, 7.20 = ⋯ = 7.20, 7, 0, 0, 0, 0)	5040.6	814.2	848.2	6702.9	-0.0061
Fairbanks	(1, 4, 6, 8, 8, 7.80 = ⋯ = 7.80, 8, 6, 1, 1, 0)	9204.4	645.3	964.4	10814.1	-0.0306

All loads are given in equivalent source energy terms, measured in kWh. Relative changes are calculated with respect to the optimal shading listed in Table 4, and their improvements from Tables 5, 6 and 7.

While window shading has a certain effect on heating and cooling loads by casting shadows on surrounding opaque wall as well, this effect is negligible compared to the impact it has by reducing solar energy incident to the window. With respect to fins, it is important to realize that they can be treated independently of each other, as they cannot both shade the window simultaneously.

The eastern fin is less pronounced in Table 4, with positions of many of its control points *P*_11_, …, *P*_14_ close to zero. Importance of the eastern fin is tested by equating positions of the control points *P*_11_, …, *P*_14_ with zero, which effectively removed the lower part of the eastern fin and replaced its upper part with a single arc from the middle of the window to the control point *P*_10_. For locations where the eastern fin is more pronounced (Miami, Houston, Phoenix, Albuquerque, Salem, Boise, Vancouver and Fairbanks), positions of the control points *P*_11_, …, *P*_14_ are also averaged, resulting in rectification of the lower part of eastern fin. Table 9 shows heating, cooling and lighting loads in equivalent source energy terms for shadings with such modified eastern fins (and already rectified overhang). Comparison with Table 8 yields that equating control points *P*_11_, …, *P*_14_ to zero increases total equivalent source energy by more than 0.1% in Miami, Houston, Phoenix, Albuquerque, Salem, Boise and Vancouver, so in these locations eastern fin may be deemed to have certain importance. Compared to the optimal shadings from Table 4 (and their improvements from Tables 5, 6 and 7), better shading from Table 9 for any location increases total equivalent source energy by less than 0.19%, and slightly decreases it in cases of San Francisco, Vancouver, Burlington and Duluth.

**Table 9:** The effects of rectifying the lower part of eastern fin.

Location	Positions of control points	*c*_*h*_*H*	*c*_*c*_*C*	*c*_*l*_*L*	*ESE*	Change (%)
Miami	(3, 7, 6, 8, 7.17 = ⋯ = 7.17, 8, 0 = ⋯ = 0)	51.6	2837.1	918.3	3807.0	1.9610
	(3, 7, 6, 8, 7.17 = ⋯ = 7.17, 8, 3.75 = ⋯ = 3.75)	57.1	2741.1	941.1	3739.4	0.1497
Houston	(4, 7, 5, 4, 2, 7.20 = … = 7.20, 1, 0 = … = 0)	760.9	1937.6	887.5	3586.1	0.5499
	(4, 7, 5, 4, 2, 7.20 = … = 7.20, 1, 4 = … = 4)	794.8	1878.0	900.1	3573.0	0.1830
Phoenix	(4, 6, 8, 6, 8, 7.40 = … = 7.40, 6, 0 = … = 0)	379.8	2505.1	911.5	3796.4	1.2783
	(4, 6, 8, 6, 8, 7.40 = … = 7.40, 6, 3.75 = … = 3.75)	399.8	2417.4	934.7	3751.8	0.0904
Memphis	(2, 2, 5, 3, 6, 6.60 = … = 6.60, 2, 0 = … = 0)	1776.5	1430.0	905.7	4112.2	0.1270
El Paso	(8, 4, 4, 2, 7, 7.67 = ⋯ = 7.67, 0 = ⋯ = 0)	891.9	1296.3	901.4	3089.5	0.0291
San Francisco	(8, 6, 5, 2, 7.67 = ⋯ = 7.67, 0, 0 = ⋯ = 0)	932.1	372.1	902.9	2207.0	-0.0717
Baltimore	(4, 7, 3, 3, 7, 7.40 = … = 7.40, 8, 0 = … = 0)	1980.7	1317.5	870.9	4169.1	0.1061
Albuquerque	(6, 6, 8, 5, 7.67 = ⋯ = 7.67, 7, 0 = ⋯ = 0)	1190.5	1457.3	863.3	3511.0	1.1282
	(6, 6, 8, 5, 7.67 = ⋯ = 7.67, 7, 4.75 = ⋯ = 4.75)	1283.7	1310.6	883.9	3478.3	0.1864
Salem	(2, 4, 3, 5, 7.33 = ⋯ = 7.33, 7, 0 = ⋯ = 0)	1974.8	767.9	899.0	3641.7	0.2616
	(2, 4, 3, 5, 7.33 = ⋯ = 7.33, 7, 2.5 = ⋯ = 2.5)	2009.8	720.7	904.8	3635.2	0.0846
Chicago	(0, 0, 3, 3, 7, 7.33 = ⋯ = 7.33, 0 = ⋯ = 0)	3376.1	1094.1	874.4	5344.6	0.0261
Boise	(4, 5, 5, 8, 7.14 = ⋯ = 7.14, 0 = ⋯ = 0)	2279.2	1095.4	894.3	4268.9	0.3117
	(4, 5, 5, 8, 7.14 = ⋯ = 7.14, 3 = ⋯ = 3)	2335.8	1018.9	902.9	4257.6	0.0460
Vancouver	(1, 5, 3, 5, 8, 7.40 = … = 7.40, 8, 0 = … = 0)	2815.3	535.3	970.7	4321.3	0.0711
	(1, 5, 3, 5, 8, 7.40 = … = 7.40, 8, 2.5 = … = 2.5)	2846.7	493.8	977.2	4317.7	-0.0122
Burlington	(0, 0, 0, 1, 6.50 = ⋯ = 6.50, 5, 0 = ⋯ = 0)	4000.0	859.9	875.2	5735.1	-0.0034
Helena	(2, 2, 4, 1, 7.17 = ⋯ = 7.17, 7, 0 = ⋯ = 0)	3201.5	862.3	875.5	4939.3	0.0534
Duluth	(0, 0, 0, 2, 6, 7.20 = … = 7.20, 7, 0 = … = 0)	5040.6	814.2	848.2	6702.9	-0.0061
Fairbanks	(1, 4, 6, 8, 8, 7.80 = … = 7.80, 8, 0 = … = 0)	9168.9	695.9	956.9	10821.7	0.0478
	(1, 4, 6, 8, 8, 7.80 = … = 7.80, 8, 2 = … = 2)	9213.5	653.9	961.3	10828.6	0.1117

All loads are given in equivalent source energy terms, measured in kWh. Relative changes are calculated with respect to the optimal shading listed in Table 4 and their improvements from Tables 5, 6 and 7.

Western fin is significantly more pronounced in optimal solutions as can be witnessed from Fig 4. Unlike the eastern fin which shades from the morning sun when both the external and internal temperatures are still low, the western fin needs to block the sun rays in afternoons when the outside temperature is close to the daily maximum. Three strategies for simplifying line of the western fin are tested here:

1. equating positions of control points *P*_0_, …, *P*_3_ to zero, which replaces the western fin with an arc located in its upper part;
2. replacing positions of control points *P*_0_, …, *P*_3_ with their average, which results in partial rectification of the western fin;
3. separately replacing positions of *P*_0_ and *P*_1_ with their average (*P*_0_ + *P*_1_)/2, and positions of *P*_2_ and *P*_3_ with the average (*P*_2_ + *P*_3_)/2, which better keeps shape of waving fins.

**Fig 6:**
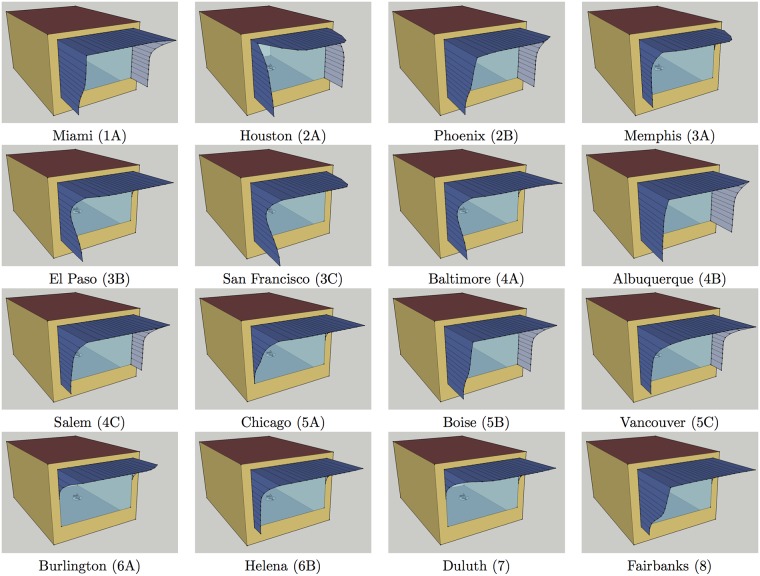
Sketchup visualisations of cellular office model with optimal simplified shading for each location.

For each location positions of the remaining control points *P*_4_, …, *P*_14_ are as in better shadings from Table 9. Table 10 gives total equivalent source energy for such modifications of western fins, while Fig 6 shows a Sketchup visualisation of the model with optimal among these shadings for each location.

**Table 10:** The effects of modifying western fin in rectified shadings.

Location	*P*_0_ = … = *P*_3_	*ESE*	Change (%)	*P*_0_ = … = *P*_3_	*ESE*	Change (%)	*P*_0_ = *P*_1_, *P*_2_ = *P*_3_	*ESE*	Change (%)
Miami	0	3849.0	3.0874	6	3741.5	0.2085	5,7	3738.5	0.1269
Houston	0	3634.8	1.9214	5	3584.5	0.5107	5.5, 4.5	3574.9	0.2422
Phoenix	0	3886.8	3.6913	6	3757.7	0.2473	5, 7	3754.8	0.1683
Memphis	0	4141.8	0.8482	3	4111.5	0.1109	2, 4	4115.4	0.2043
El Paso	0	3153.0	2.0840	4.5	3094.8	0.2003	6, 3	3091.0	0.0765
San Francisco	0	2244.2	1.6129	5.25	2211.7	0.1410	7, 3.5	2208.8	0.0097
Baltimore	0	4226.0	1.4720	4.25	4168.9	0.1019	5.5, 3	4168.7	0.0959
Albuquerque	0	3639.2	4.8211	6.25	3478.1	0.1818	6, 6.5	3477.2	0.1545
Salem	0	3680.0	1.3163	3.5	3637.1	0.1359	3, 4	3636.0	0.1051
Chicago	0	5350.4	0.1344	1.5	5347.2	0.0739	0, 3	5344.6	0.0261
Boise	0	4326.8	1.6734	5.5	4261.5	0.1394	4.5, 6.5	4258.5	0.0671
Vancouver	0	4354.8	0.8471	3.5	4319.6	0.0316	3, 4	4318.7	0.0096
Burlington	0	5735.8	0.0077	0.25	5738.1	0.0476	0, 0.5	5736.4	0.0182
Helena	0	4955.0	0.3705	2.25	4939.2	0.0502	2, 2.5	4938.5	0.0376
Duluth	0	6703.3	0.0005	0.5	6710.4	0.1059	0, 1	6706.2	0.0437
Fairbanks	0	10857.7	0.3805	4.75	10831.8	0.1404	2.5, 7	10822.4	0.0536

Total equivalent source energy for heating, cooling and lighting loads is given in kWh. Positions of the remaining control points *P*_4_, …, *P*_14_ for each location are as in a better shading from Table 9. Relative changes are calculated with respect to the optimal shading listed in Table 4 and their improvements from Tables 5, 6 and 7.

It is easily seen from Table 10 that the western fin is more important than the eastern fin, as equating positions of control points *P*_0_, …, *P*_3_ to zero may increase total equivalent source energy by up to 4.82% (in Albuquerque). The western fin may be deemed irrelevant in Burlington and Duluth only, where this increase is less than 0.01%. Comparing with the optimal shadings from Table 4, and their improvements from Tables 5, 6 and 7, it can be seen the shadings, with the overhang, eastern and western fins simplified in this way, are no longer better than those found by genetic optimisation. However, increase in total equivalent source energy obtained by simplifying design is at most 0.24% in Houston, while in San Francisco, Vancouver and Burlington it is less than 0.01%. Moreover, visualisations of optimal simplified shadings in Fig 6 clearly show that their outer edges are less winding when compared to shadings from Fig 4.

## Conclusion

An important finding of this study is that, although genetic optimisation was given wide freedom in selecting design of outer edge of shading for a cellular office in representative USA climates, the overhang control points in the resulting optimal solutions all had relatively similar positions. This at least partially rejects the initial expectation that curvilinearity of solar paths should induce curvilinearity of the optimal shading shape, as the overhang in optimal shading shapes is close to being rectangular. Another important finding is that the structure of shading shape can be significantly simplified with only a slight increase of up to 0.24% in total equivalent source energy for heating, cooling and lighting loads compared to the optimal shape found by genetic optimisation. Hence instead of 15 control points, with seven control points defining the overhang and five control points for each of the fins (with two common control points for joints of the overhang with the fins), one can achieve very similar results by identifying depths of successive control points, thus dividing the shading into six independent regions: the lower and the upper part of the western fin, joint of the western fin and the overhang, interior part of the overhang, joint of the eastern fin and the overhang, and the whole eastern fin of a shading in a cellular office, although this smaller number of regions requests higher resolution of their feasible depths. This opens up an opportunity to use depths of these regions, together with other parameters such as the window size, glazing properties, and climate characteristics, in a regression analysis in future work and possibly get explanation for the appearance of holes between overhang and fins in the optimal shading for Houston and protrusion of the lower part of western fin in optimal shadings for El Paso, San Francisco and Baltimore.
